# Gestational and lactational exposure to HIV tri-combination therapy induces sex- and dose-dependent changes in inflammatory cytokine profiles, intestinal permeability, and villi morphology in adult rat offspring

**DOI:** 10.3389/fimmu.2026.1668334

**Published:** 2026-03-11

**Authors:** Yaswanthi Yanamadala, Kuppan Gokulan, Kumari Karn, Vicki Sutherland, Helen Cunny, Janine H. Santos, Kelly Davis, Sangeeta Khare

**Affiliations:** 1 Division of Microbiology, National Center for Toxicological Research, US Food and Drug Administration, Jefferson, AR, United States; 2 Division of Translational Toxicology, National Institute of Environmental Health Sciences, Research Triangle Park, NC, United States; 3 Toxicologic Pathology Associates, Jefferson, AR, United States

**Keywords:** cytokine, gene expression, gestational exposure, HIV, intestinal permeability

## Abstract

**Introduction:**

Gestational antiretroviral therapy (ART) has significantly reduced the risk of vertical transmission of HIV, but concerns linger about its long-term effects on the fetal immune system and intestinal health. Our previous work has demonstrated dose-dependent changes in the fecal and mucosa-associated microbiome of adult rat offspring perinatally exposed to TC-ART (tri-combination ART: dolutegravir, abacavir, and lamivudine). These changes may either be driven by alterations in immune system and intestinal barrier integrity or potentially impact them.

**Methods:**

In this study, we further investigated the long-term effects of perinatal TC-ART exposure on intestinal permeability, cytokine profiles, and intestinal mucosa morphology.

**Results:**

We observed statistically significant sex-dependent differences, with male offspring exhibiting reduced weight gain, a dichotomous response between low and high dose for inflammatory cytokines [interleukin-5 (IL-5), IL-7, and IL-12], differential regulation for the mRNA expression of intestinal permeability-related genes (21 downregulated), and disrupted villous architecture, while females showed dose-dependent decreases in inflammatory cytokines [IL-17, IL-5, and macrophage colony-stimulating factor (M-CSF)]. In females, while some intestinal permeability genes were downregulated, the upregulation of other permeability genes suggests a compensatory mechanism to maintain the intestinal barrier function, indicating an overall milder response to TC-ART.

**Discussion:**

These findings suggest that perinatal exposure to TC-ART may have differential impacts on intestinal health, with females exhibiting a more adaptive response compared to males, highlighting the need for sex-specific considerations in evaluating long-term effects of ART.

## Introduction

Antiretroviral therapy (ART) during pregnancy is crucial to prevent mother-to-child transmission (MTCT) of HIV; however, there are several concerns about the potential adverse effects on the fetus associated with ART, including metabolic, cardiac, and neuronal abnormalities (1–3). These effects are influenced by several factors, including the type of ART, fetal exposure levels, and timing of dose initiation during pregnancy. Tri-combination ART (TC-ART), containing dolutegravir (DTG), abacavir (ABC), and lamivudine (3TC), is often prescribed after organogenesis when its benefits such as effective viral suppression (93%) (4) and MTCT (<3%) (5) outweigh the potential risks, particularly when alternative regimens are less effective. When started before conception, DTG-based regimens are shown to reduce MTCT to almost 0% (6). However, the long-term effects of this potential drug regimen on the fetus, particularly on the gut, which is recognized as a cornerstone for the overall health, remain unexplored.

The intestinal barrier is a multifaceted system composed of several protective layers including the outer mucosal barrier, epithelial cells, and the functional immunological barrier including Peyer’s patches (7). The mucus layer serves as a habitat for numerous commensal bacteria and coordinates with epithelial cells to maintain barrier integrity and defense. Disruption of these barriers has been linked to the development of several immune, respiratory, neurological, and cardiovascular conditions (8–10). The gastrointestinal tract possesses the largest component of the immunological tissue in the body, and it plays a key role in shaping the overall health by maintaining a symbiotic relationship with the commensal gut microbiota (11). There is mounting evidence of the involvement of proinflammatory molecules like interleukin-6 (IL-6), IL-8, IL-18, and tumor necrosis factor-α (TNF-α) in the progression of several diseases (12).

Newborns are considered to be sterile at birth, and the microbiota is expected to develop through exposure to surrounding environments, dietary intake, and mother (8). The early colonization of the gut microbiome plays a crucial role in developing an infant’s immune system and overall gut health, highlighting the influence of maternal health on that of the offspring. It has been shown that the route of xenobiotic exposure (oral or intravenous) can differentially impact the intestinal responses of the recipient (13). However, little is known regarding the potential impact of *in utero* exposure of a drug or xenobiotic in adult offspring. Independent studies in human patients have shown that persistent HIV infection despite the treatment with ART can cause gut mucosal immune alterations, intestinal barrier damage, and gut microbial changes (14, 15). Similar disruptions, including gut-associated lymphoid tissue (GALT) that cause CD4^+^ T-cell depletion, have been reported in an uninfected offspring born to HIV-infected individuals (16).

The changes in the intestinal mucosa permeability or gut mucosa-associated immune profiles have been shown as some of the contributing factors for microbial translocation and microbial alterations, which could lead to several adverse outcomes (17). It is becoming more evident that HIV-exposed uninfected children are highly susceptible to infectious diseases (18). It is unclear if these infections could be due to the residual viral load or long-term effects of ART exposure during early development. To help address questions such as these, this study was conducted in the adult offspring of healthy animals exposed to TC-ART during gestation. Previously, we reported the effects of TC-ART on fecal and mucosa-associated microbial abundance in perinatally exposed aged rat offspring (both male and female). In this study, we investigated the effects of *in utero* exposure to TC-ART on intestinal permeability, inflammatory cytokines, and the morphology of the intestinal tissue to assess the intestinal health of the offspring.

## Materials and methods

### Animals

The experimental procedures, animal housing, and treatment protocols for this study were the same as those described in our previous work (19, 20). Time-mated Sprague–Dawley (Hsd: SD) rats were obtained from Envigo (Indianapolis, IN). All animals (pregnant female rats and their male and female offspring) were housed in the animal facility at Amplify Bio, West Jefferson, OH, an independent scientific contract research organization. Time-mated rats were approximately 11–14 weeks of age upon receipt. The facility’s Institutional Animal Care and Use Committee (IACUC) reviewed the protocol and approved it (IACUC protocol T06055). Animals were housed in polycarbonate cages with irradiated hardwood bedding chips (Sani Chips^®^; Envigo, Madison, WI). During gestation and lactation, rats were provided natural crinkled kraft paper for enrichment (Crink-l’nest™, The Andersons; Maumee, OH). Offspring remained with their respective dams until postnatal day (PND) 21. After weaning, F1 offspring were provided polycarbonate rectangular shelters (Rat Retreats™, Bio-Serve; Flemington, NJ) as enrichment and were group housed by sex, up to five per cage. During gestation and lactation, animals were fed irradiated NIH-07 pellets or wafers (Zeigler Bros., Gardners, PA). After weaning, animals were fed NTP-2000 (Zeigler Bros., Gardners, PA). Rats were provided municipal water *ad libitum* from an automatic watering system; both water and feed were analyzed for known contaminants that could interfere with or affect the outcome of the study, and none were found.

### Dose selection and administration

Rats were randomly assigned to one of three groups: Control (vehicle: 0.2% methylcellulose/0.1% Tween 80), low dose (LD) (150/12.5/75 mg/kg ABC/DTG/3TC), or high dose (HD) (300/25/150 mg/kg ABC/DTG/3TC). Body weights were measured every 3 days, and the doses were adjusted to the body weight (dosing volume, 5 mL/kg). Maternal rats were dosed via oral gavage starting on gestational day (GD) 6 and continued through PND 21. The offspring in the study were only exposed to the drug indirectly via maternal transmission and did not receive the drug directly. The offspring remained with their dams until weaned on PND 21, after which one male and female offspring from each litter were selected for the study. These offspring (*n* = 5 for each group per sex) were used for assessments at 12 months of age. [Note: these animals were a subset of a larger study published earlier (19, 20).] Animals were euthanized using carbon dioxide (CO_2_) delivered from a compressed gas cylinder using a flow rate that was appropriate for the chamber type and size and in accordance with the AVMA guidelines. Gas flow was maintained for at least 1 min following apparent death, as indicated by cessation of movement and respiration. Death was confirmed using a secondary physical method, in accordance with AVMA recommendations.

### Assessment of body weights

The offspring weights were collected from the day of weaning until the study termination on day 365.

### Cytokine detection in the ileal tissue

Following IACUC-approved protocols, the animals were euthanized using carbon dioxide. Ileal tissue was carefully collected adhering to sterile conditions and flushed using sterile phosphate-buffered saline (PBS) to remove fecal material. The tissue samples were then snap-frozen in liquid nitrogen and shipped to NCTR (National Center for Toxicological Research), Jefferson, AR, for subsequent analysis.

#### Protein extraction and quantification of cytokines from ileal tissue

The ileal tissues (equal to 9 mm biopsy punch) collected from the offspring were homogenized using protein lysis solution (Bio-Rad, Hercules, CA) in gentleMACS™ M tubes and a gentleMACS dissociator (Miltenyi Biotec, Auburn, CA), with intermittent homogenization cycles (1 min homogenization, 1 min on ice, repeated twice). The protein lysate was then centrifuged at 700×*g* to remove non-homogenized tissue and 10,000×*g*, consecutively and a clear homogenate was quantified for protein concentration using the Bio-Rad protein assay reagent (Bio-Rad, Hercules, CA). The clarified ileal tissue homogenate was diluted to a protein concentration of 900 µg/mL, and 50 µL of the lysate was used for the cytokine assay. The Bioplex rat cytokine 23-plex panel kit (Bio-Rad, Hercules, CA) was used to assess the cytokine levels in the intestinal tissue and detected using the Bio-Rad Bioplex instrument as described earlier (21).

### mRNA expression of intestinal mucosal cell–cell junction permeability-related genes

#### RNA extraction from the ileal tissue and cDNA conversion

RNA extraction and cDNA conversion were conducted as previously described in Gokulan et al. (22). The ileal tissue was homogenized using a handheld homogenizer in Trizol reagent (Fisher Scientific, Hanover Park, IL) followed by organic extraction with chloroform. The samples were centrifuged at 10,000×*g* to separate the aqueous layer. Equal proportion of isopropanol (Sigma Aldrich, St Louis, MO) was added to precipitate the RNA and centrifuged at 15,000×*g* after overnight incubation at −20°C. The pellet was washed with 70% ethanol and resuspended in nuclease-free water. To remove the genomic DNA contamination, equal amounts of RNA [detected by BioTek Cytation instrument (Agilent Technologies, Santa Clara, CA)] was taken from each sample and treated with DNase (Fisher Scientific, Hanover Park, IL). Then, 2.5 µg of pure RNA was converted to cDNA using the Thermo Fisher Superscript IV VILO Kit.

#### Quantification of expression of permeability-related genes

The cDNA was used for RT-PCR to detect the transcription levels of 84 genes that are involved in maintaining the intestinal permeability using the PARN-213Z Rat cell junction pathfinder kits (Qiagen LLC, Germantown, MD). SYBR Green containing Master mix (Qiagen LLC, Germantown, MD) was used to assess the fluorescence. The run conditions were 95°C for 10 min followed by 95°C for 15 s and 60°C for 1 min for 40 cycles. The run was performed and detected by Bio-Rad CFX384 RT-PCR (Bio-Rad, Hercules, CA) instrument.

### Histopathological findings

For the assessment of any morphological changes, light microscopic evaluation of hematoxylin and eosin (H&E)-stained and Gram-stained sections of ileum from rats was conducted. A 2- 3-cm length of ileum was fixed in 10% neutral buffered formalin (NBF) for 24 h, transferred to 70% ethanol, and processed to paraffin-embedded blocks. The paraffin-embedded tissue sections of ileum were sectioned at 4–6 µm thick and mounted on glass slides. One tissue section from ileum of each animal was stained with H&E, and a replicate section was Gram-stained. The H&E-stained and Gram-stained sections of ileum from each animal were evaluated by light microscopy. The subjective evaluation of the grading of villus atrophy was done after the H&E-stained slides were scrambled (randomly mixed) and read “blind” to the animal number and treatment group. All microscopic findings were recorded in a Microsoft Excel spreadsheet. The grading of the minimal or mild severity of villus atrophy was evaluated subjectively based on the combination of several factors: (i) the severity of a decreased height of the villi; (ii) the severity of an increased thickness of the villi; and (iii) the severity of the proportion of villi that appeared to be fused with one or more other villi. Animals with mild villus atrophy had more villi affected with the above factors with a greater severity of decreased height and increased thickness than animals with minimal villus atrophy.

### Statistical/data analysis

#### Statistical analysis of the cytokine data

The data for the cytokines were analyzed using the Bioplex manager and Bio-Plex Data pro software. Depending on the data distribution and variance, either a Welch’s *t*-test or analysis of variance (ANOVA) was performed. Only cytokines demonstrating statistically significant differences between the treatment and control groups are reported in the Results section to prevent overinterpretation of non-significant findings.

#### Statistical analysis of qPCR data using the Gene Globe software

The obtained CT values were organized, and data for all experimental conditions were exported as an Excel file. The data for the permeability (mRNA expression of genes involved in the cell–cell junctions) were analyzed using the gene globe software (Qiagen, https://geneglobe.qiagen.com/). β-actin was used as the housekeeping gene. A parametric, unpaired, Student *t*-test (assuming equal variances) and a two-tailed distribution were used to calculate the significance. Fold changes greater than 2 and *p* ≤ 0.05 were considered significant. Treatment groups LD and HD, represented as fold regulations, change compared to the control group (normalized to 1). Only genes showing statistically significant changes are presented in the Results section.

## Results

The aim of this study was to evaluate the long-term effects of indirect TC-ART exposure on the intestinal health of rat offspring. A 12-month-old adult rat is comparable to a 30-year-old human, providing a relevant comparison for studying long-term outcomes (23). Treated groups were compared to non-exposed, vehicle-treated control animals (both male and female) to assess sex-specific changes resulting from the drug exposure during early developmental stages.

### Dose-dependent effects of TC-ART on the average weight gain in male offspring

**Figure 1:**
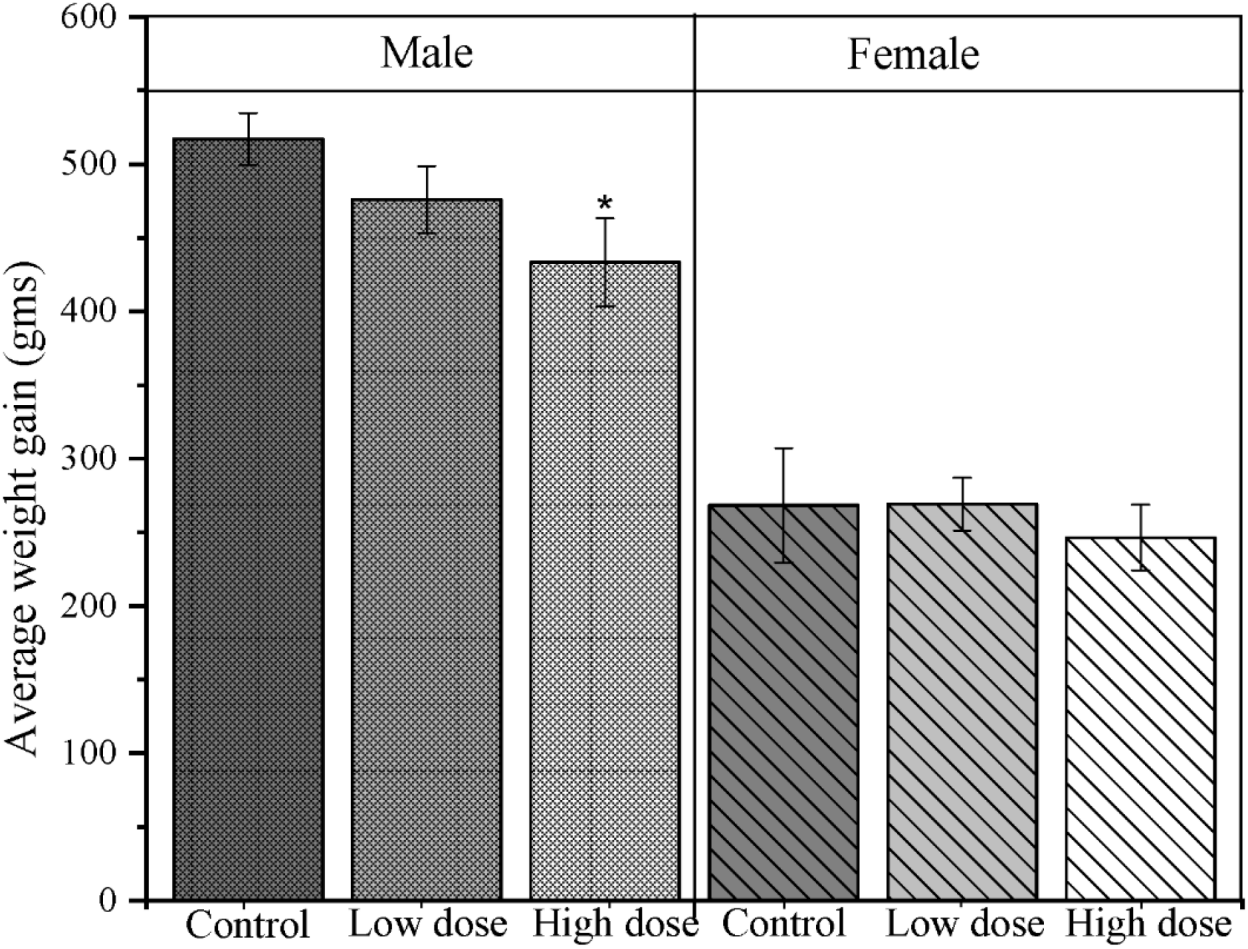
Average weight gain in adult offspring following gestational and lactational TC-ART exposure: Altered average weight gain in adult male and female offspring perinatally exposed to TC-ART across treatment groups. The bar graph showing the average weight gains (*n* = 5 offspring/sex/group) in grams of control, low dose (LD), and high dose (HD). Error bars indicate mean ± SEM. Statistical comparisons were performed within sex using one-way ANOVA (*p < 0.05). Male animals showed consistent decreased weight gain compared to female animals.

The average weight gain from PND 22 to PND 365 was analyzed between the groups. The exposed male animals showed a dose-dependent decrease in average weight gain compared to the control animals. HD male offspring had an average weight that was 83.78% of the control group, reflecting a 16.22% reduction. In contrast, female offspring were minimally affected, showing only an 8.14% decrease in average weight gain compared to controls (**Figure 1**). These findings suggest that the HD might have negatively affected weight gain in male and possibly female animals over the study period, with more pronounced effects on male animals.

### Sex- and dose-dependent impact of TC-ART on offspring ileal cytokines

**Figure 2:**
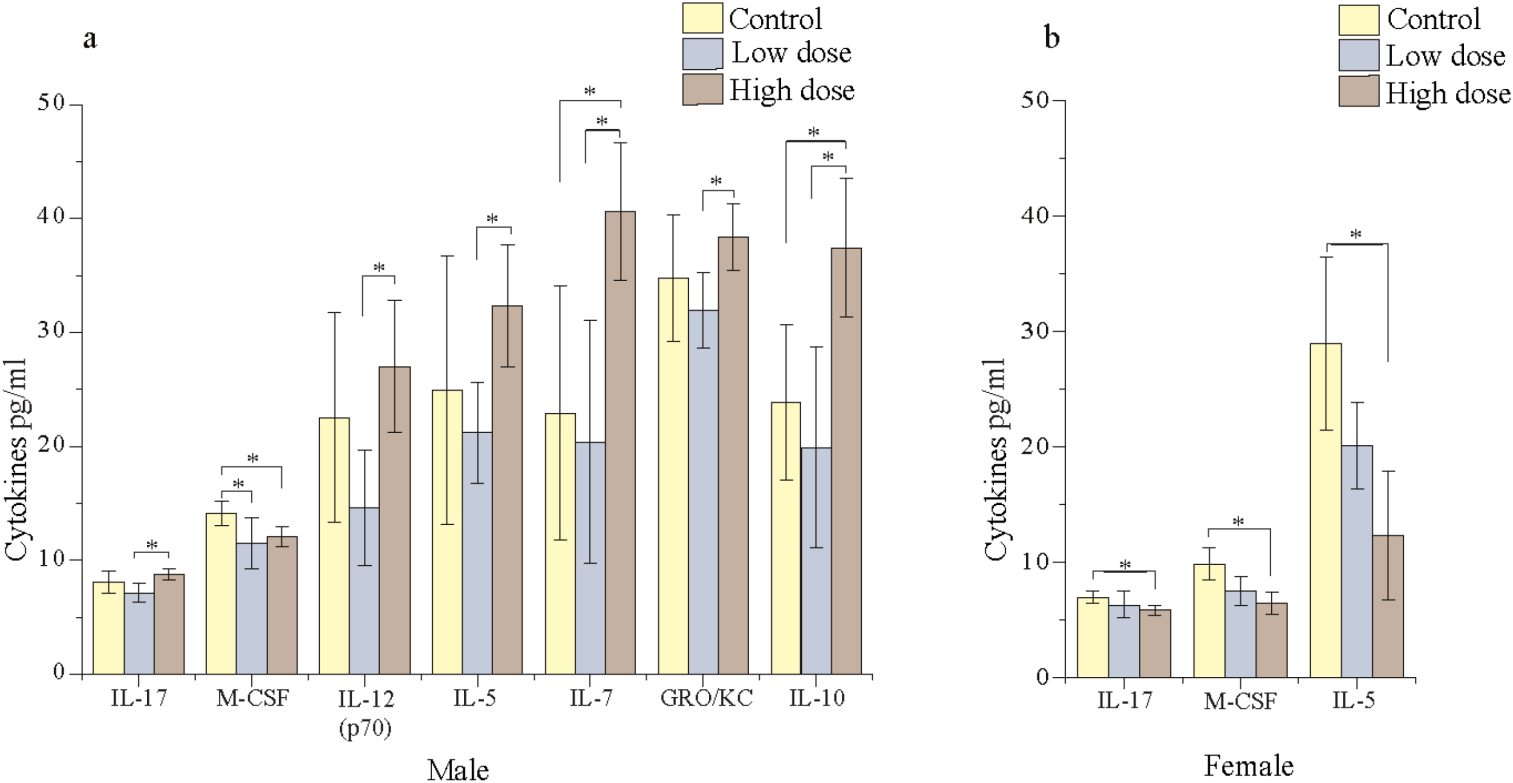
Ileal cytokine profiles in adult offspring exposed to TC-ART: Sex and dose-dependent alterations in ileal cytokine levels. Increased levels of selected proinflammatory cytokines in TC-ART-exposed offspring. Only cytokines demonstrating statistically significant differences between the treatment and control groups are reported here. **(a)** Male offspring showed significant changes in pro- and anti-inflammatory cytokine levels (IL-10), chemokines (GRO/KC), cell proliferation/growth factor (M-CSF), proinflammatory cytokines [IL-17, IL-12(p70), IL-5], and immune cell development factor (IL-7) across doses. **(b)** Female animals showed fewer cytokine alterations, with significant changes observed in three cytokines. Data represent mean ± SEM (*n* = 5/sex/group); statistical significance was determined by one-way ANOVA (*p < 0.05).

The cytokine changes in the offspring were assessed using the multiplex immunoassay. The effects of the TC-ART on the male offspring showed differential responses compared to the female offspring (**Figures 2a, b**). Male offspring exposed to both the HD and LD showed a greater number of changes in cytokine expression profiles compared to the females. While female animals displayed dose-dependent reductions in the cytokine levels, male offspring showed a reduction with LD exposure but an increased expression with HD exposure. Notably, the proinflammatory cytokines macrophage colony-stimulating factor (M-CSF), IL-17, and IL-5 were significantly altered in both male and female groups; in males, although neither dose showed a significant difference from the control, the marked reduction at the LD and increase at the HD resulted in significant differences in the two treatments (**Figures 2a, b**). In females, these cytokines decreased with HD exposure, further emphasizing a sex-specific response. In males, except for the anti-inflammatory IL-10 and chemokine [CXCL1 (GRO/KC)], most of the remaining cytokines that were significantly altered are proinflammatory cytokines, while in females, all three cytokines that were significantly altered were proinflammatory (**Figure 2b**). Except for IL-7, a proinflammatory cytokine, all other proinflammatory cytokines that were reduced with the LD showed levels comparable to those of the vehicle control animals. In contrast, IL-7 expression was markedly upregulated with the HD in males.

### Perturbation of cell-permeability-related genes due to gestational and lactational exposure to TC-ART

**Figure 3:**
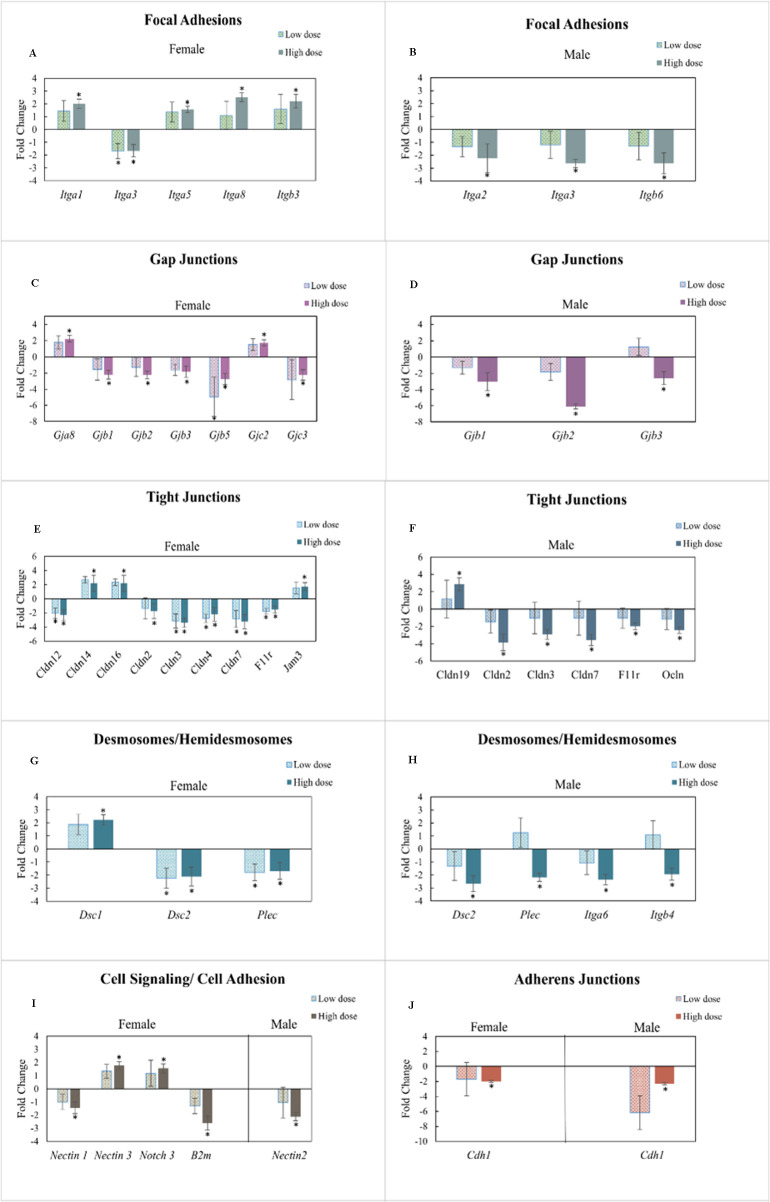
Ileal mRNA expression of permeability-related genes in adult offspring perinatally exposed to TC-ART. **(A, C, E, G)** represent female offspring, while **(B, D, F, H)** represent corresponding data for male offspring (**I, J** include both sexes). Genes are grouped by functional categories of cell junction genes, including focal adhesion **(A, B)**, gap junction **(C, D)**, tight junctions **(E, F)**, desmosomes/hemidesmosomes **(G, H)**, cell signaling/cell adhesion **(I)**, and adherens junctions **(J)**. Data normalization was performed using the housekeeping gene *Actb* (β-actin). Treatment groups—low-dose and high-dose —represented as fold regulation change compared to the control group (normalized to 1). Only genes showing statistically significant changes are presented here. Data are expressed as mean ± SE. *p* < 0.05 was considered statistically significant compared to control.

The intestinal permeability of the offspring for both male and female animals showed significant perturbations in the expression of intestinal epithelial cell integrity-related genes (**Figure 3**). Unlike the cytokines, there was an increased number of altered genes in female compared to male animals. Out of 84 key cell-permeability-related genes, the TC-ART gestational and lactational exposure contributed to 45% of altered gene expression in both males and females across both doses. LD-exposed male offspring did not show significant changes in the expression of permeability-related changes. On the other hand, the female animals exposed to the HD showed upregulation of focal adhesion genes (**Figure 3A**) and the HD-exposed males showed downregulation of the focal adhesion genes (**Figure 3B**). Most of the gap junction genes were downregulated in both male and female animals (**Figures 3C, D**) while a few of them, including *Gja8* and *Gjc2* (*Gjd2* not significant), were upregulated in females (**Figure 3C**). Expression of Tight junction-related genes [*Claudins* (*Cldn*) and *Occludins* (*Ocln*)] was mainly downregulated in both male and female (**Figures 3E, F**). The HD-treated females showed the upregulation of *Cldn14*, *Cldn16*, and *Jam*3 genes and the downregulation of only one gene (*Cldn19*). The tight junction genes in males were downregulated in both the LD and HD; however, statistical significance was only observed in the HD (compared to the vehicle controls). mRNA expression of desmosomes/hemidesmosomes also showed differential expression of some common and unique genes in female and male offspring (**Figures 3G, H**). Expression of *Dec2* and *Plec* was downregulated in the LD as well as the HD in female, and statistically significant differences were observed in HD-treated males as compared to control. In males, the HD also caused downregulation of *Itga6* and *Itga4*. The transcription of a greater number of cell signaling/cell adhesion-related genes was differentially impacted between the sexes (**Figure 3I**). Expression of *Cdh1*, an adherens junction gene, was downregulated in the HD-exposed male and female offspring, but the effects in the males were more pronounced (**Figure 3J**). Overall, there was a sex- and dose-dependent impact of early developmental exposure to TC-ART on the adult offspring. Moreover, comparatively, there were fewer changes in mRNA expression of permeability-related genes in the LD- relative to the HD-exposed animals.

### Morphological changes in male and female offspring due to gestational and lactational exposure to TC-ART

The transcriptional changes reported above, including that of cell–cell junction-related genes, may lead to dysfunctional intestinal barrier and morphological changes in the villous structure. Thus, histopathological assessment of the intestinal mucosal tissue was conducted to obtain an in-depth understanding of the dose- and sex-based responses of TC-ART.

#### Neoplastic findings

There were no neoplastic findings in this portion of the study.

#### Non‐neoplastic findings

**Figure 4:**
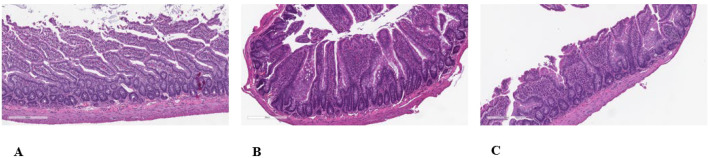
Histologic evaluation of intestinal sections of the ileal mucosa and submucosa of male offspring exposed to TC-ART *in utero*. Normal histologic appearance of absorptive villi of H&E-stained sample for control male **(A)**. Minimal to mild villar atrophy was observed in HD-exposed TC-ART *in utero* [**(B, C)**, respectively]. Features of atrophy included blunted villi of reduced height and increased width due to an increased amount of villar interstitial tissue often with partial or complete fusion of two or more adjacent villi.

**Figure 5:**
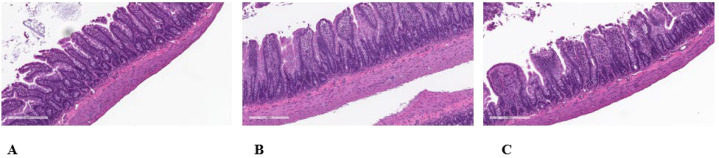
Histologic evaluation of intestinal sections of the ileal mucosa and submucosa of female offspring exposed to TC-ART *in utero*. Normal histologic appearance of absorptive villi of H&E-stained sample for control female **(A)**. Minimal to mild villar atrophy was observed in HD-exposed TC-ART *in utero* [**(B, C)**, respectively]. Features of atrophy included blunted villi of reduced height and increased width due to an increased amount of villar interstitial tissue often with partial or complete fusion of two or more adjacent villi.

The hallmark of the histopathology was a discrete difference in villous atrophy in male offspring compared to concurrent vehicle control males that was associated with the perinatal exposure to TC-ART. The villous atrophy consisted of villi with reduced height but increased width, which showed increased in incidence and average severity in male rats (**Figures 4A–C**). The crypts often appeared to be of reduced depth (**Figures 4B, C**). Often there appeared to be a partial or complete fusion of two or more adjacent villi. Villi with increased width often appeared to have an increased amount of villar interstitial tissue. However, there are several reported diagnostic features of villar atrophy that were not present in this study, namely, a reduced mitotic rate; a reduced number of mucous (goblet) cells; increased cell loss through apoptosis or necrosis; loss of the normal arrangement of the epithelium; and/or the absence of flattened, cuboidal, or vacuolated epithelial cells. In females, however, a relationship between *in utero* exposure to the TC-ART and villi changes was unclear (**Figures 5A–C**). The slight increased incidence of the finding in the female offspring of HD-treated females compared to the female offspring of vehicle control females suggests that the villous atrophy may be related to the *in utero* exposure to the TC-ART. However, the decreased severity of the finding in affected female offspring from LD- and HD-treated females compared to the severity of the finding in female offspring of untreated vehicle control females suggests that the villous atrophy in female may not be related to *in utero* exposure to the TC-ART.

The submucosa, submucosal and myenteric nerve plexi, tunica muscularis, and the subjective assessment of the number of goblet cells and Paneth cells in the mucosa were considered normal in the ileum of all 30 animals. The prevalence of microscopic findings is summarized in **Table 1**. Autolysis of the tips of the villi in the ileum was not considered related to *in utero* exposure to the TC-ART (**Figure 5**) as it consisted of loss/sloughing of cells at the tips of the villi with no inflammatory reaction and was present in all concurrent vehicle control animals. This is typically caused by extremely rapid post-necropsy preferential loss/sloughing of the cells through the actions of the cells’ own enzymes, although it can also occur from handling artifacts.

**Table 1:** Prevalence of microscopic findings in the ileum of male and female offspring from pregnant Sprague–Dawley rats that were untreated controls or treated during gestation with a low dose (LD) or high dose (HD) of TC-ART.

Tissue	Diagnosis (villous atrophy)	Male			Female		
		Untreated control	Low dose	High dose	Untreated control	Low dose	High dose
Small intestine, ileum	Diagnosis count	1	5	5	3	3	4
	Number of animals examined	5	5	5	5	5	5
	Diagnosis %	20%	100%	100%	60%	60%	80%
	Average severity	1	1.4	1.8	1.7	1	1.5

## Discussion

The use of DTG-based regimens for HIV treatment, particularly in pregnant women, has dramatically reduced the MTCT (6). Given that TC-ART drugs could exhibit anti-bacterial properties (24), and influence the immune profiles (25, 26), they may alter the maternal gut homeostasis. Furthermore, since the drug is absorbed through the small intestine (27), maternal exposure during gestation and lactation may plausibly influence intestinal and immune development that have implications on long-term consequences on the offspring gut health.

Our previous manuscript using the same animals demonstrated that TC-ART exposure had the potential to disrupt intestinal microbial profiles in the offspring upon indirect exposure through the mothers (19, 28). As the literature suggests a strong interplay between the gut microbiome, immune signaling, intestinal barrier permeability, and functioning (22, 29–31), it is crucial to assess whether disruption in microbial profiles extend to changes in weight gain, intestinal permeability, cytokine expression, and morphological alterations in the ileal mucosa. Our results align with our previous findings (28) where we reported sexually dimorphic responses. Specifically, in this study, we found that adult males perinatally exposed to the HD exhibited more pronounced changes than females, including increased cytokine alterations, downregulation of cell–cell junction-related genes, significant ileal morphological changes, and reduced body weight at the HD. Interestingly, females not only exhibited indicators of immune suppression but also showed compensatory epithelial responses. While both males and females exhibited changes in the ileal mucosa-associated responses (such as changes in permeability, inflammatory cytokines, and villous morphology), the mechanisms associated with the perinatal TC-ART exposure appear distinct with males showing inflammatory regulation and structural damage (villous atrophy) that is dependent on the dose/treatment. A key observation was the reduction in proinflammatory cytokines (IL-12, IL-7, and GRO/KC) in males exposed to the LD, which was not observed in females. Most notably, proinflammatory cytokines were significantly increased in the HD-exposed males relative to the LD, although these changes were not significant relative to those of vehicle controls. These data suggest a dose-dependent effect in immune modulation. The increased expression of IL-10 (anti-inflammatory) at the HD may indicate a compensatory mechanism to restore immune homeostasis. Similarly, IL-7, which is crucial for lymphocyte survival and gut barrier maintenance, was also upregulated. Notably, accumulation of IL-7 in intestinal epithelial cells has been suggested as a survival mechanism adapted to limit the progression of inflammatory and metabolic diseases (32). IL-5, which is associated with eosinophil activity and gut homeostasis, was significantly altered in both males and females; however, the reduced IL-5, M-CSF, and IL-17 (33) in females could have contributed to altered epithelial integrity. A previous study showed that reduced IL-17 caused changes in the gut microbiota and increased pathogenic bacteria (34). The decreased IL-17 in the exposed animals could plausibly contribute to the previously reported altered microbiome in the same animals (20). IL-5 and M-CSF showed dose-dependent reduction in both male and female animals. These cytokine disruptions, particularly downregulation of IL-5, M-CSF, and IL-17, may impair mucosal immunity and epithelial repair. Several of the cytokines that were altered in the males (IL-12, IL-17, M-CSF, and IL-10) are often linked to metabolic disruption, and these changes could have contributed to the weight loss in male offspring (35). Although there is limited literature to suggest a relationship between these cytokines and intestinal barrier integrity, it is plausible to hypothesize that IL-5 and M-CSF may have contributed to the intestinal barrier changes.

Although specific cell junctions predominate in certain cell types, all cells interact with their environment via multiple types of cell–cell junctions. The focal adhesions and adherens junctions connect intracellular domains of cell surface receptors to actin filaments, while desmosomes and hemidesmosomes connect to intermediate filaments. The downregulation of focal adhesion genes, such as integrins (*Itga2*, *Itga3*, and *Itgb6*), in males indicates transcriptional alterations in pathways involved in cell–matrix connections, consistent with prior reports linking these genes to epithelial stability (36, 37).

Adjacent cells communicate through gap junctions that allow the passage of molecules, ion, and electrical impulses between them. Downregulation of *Gjb1*, *Gjb2, Gjb3, Gjb5*, and *Gjc3* (in females) and *Gjb1*, *Gjb2*, and *Gjb3* (in males) may indicate decreased activity or regulation of gap junction-related pathways involved in intercellular communication. However, in females, upregulation of *Gja8* and *Gjc2* suggests differential regulation of cellular communication between adjacent cells.

The differential expression of tight junction genes (claudins, occludins, and junctional adhesion molecules) in females compared to males could reflect sex-specific regulation barrier associated pathways in response to the structural alterations. Claudins are crucial regulators of intestinal permeability (30, 38), and their downregulation in both males and females has been associated with altered paracellular transport. The significant morphological changes (villous atrophy) in male offspring align with the downregulation of claudins (*Cldn2*, *Cldn3*, *Cldn4*, and *Cldn12*), suggesting coordinated structural and transcriptional alterations in barrier integrity-related pathways, potentially facilitating microbial translocation and significant shifts in bacterial community (previously reported) and further immune activation as seen in cytokine profiles (19, 20, 28). The downregulation of *Cldn7* observed in both male and female animals is often seen in inflammatory conditions (39, 40). Changes in claudin expressions (tight junction related genes) are linked to metabolic disorders (41), and the downregulation of these genes may contribute to reduced weight in males compared to females. The downregulation of adherens junctions leads to the disruption in the barrier function (42). The balance between these processes is important in maintaining the selective barrier permeability, which seems to be compromised by the significant alteration of 45% of the genes and several other genes showing a trend of changes.

Increased intestinal permeability as suggested by downregulation of cell–cell junction-related genes may have led to flattening or shortening of the villi, reducing the absorptive surface area, and thus leading to malabsorption. Moreover, systemic inflammation and increased presence of pathogenic bacteria along with the reduced absorption of nutrients could impact the weight of the animals. In contrast, female offspring, despite the morphological changes in the villi, did not show significant weight deficits. This could be due to preserved tight junction integrity or compensatory mechanisms (reduced proinflammatory molecules and upregulated adhesion genes and integrins) or improved microbial diversity (19) and reduced number of pathogenic bacteria that could help maintain the nutrient absorption.

The microscopic finding of villous atrophy in the ileum of male rats was considered related to *in utero* exposure to the TC-ART showing villar blunting, fusion, and thickening. However, the relationship of villous atrophy in the ileum of female rats to the *in utero* exposure to the TC-ART was unclear under the conditions of this study. Villous atrophy has been reported to be induced by the withdrawal of food and the administration of antimitotic cytotoxic agents, secondary to chronic inflammation or following rotavirus infection (43). IL-5, IL-7, IL-17, M-CSF, IL-10, and IL-12 play a crucial role in gut homeostasis and epithelial turnover, and their dysregulation may contribute to villous atrophy. M-CSF is essential for epithelial repair (44), and its downregulation has been associated with impaired mucosal healing responses, which may relate to epithelial damage and villous fusion. While IL-7 is involved in maintaining epithelial integrity (32), excessive or dysregulated expression as seen in inflammatory conditions can disrupt epithelial architecture. In males, M-CSF was reduced, but the increased levels of IL-7, IL-10, IL-12, and GRO/KC suggest increased immune activation. This, in turn, could drive low-grade inflammation and epithelial turnover imbalance, leading to villous atrophy. A combination of immune activation and altered barrier-associated gene expression in males may have more pronounced response than the immune suppression seen in females.

This pattern of sex-specific differences in gut structure and immune signaling is consistent with clinical reports of ART drug toxicity in HIV-exposed but uninfected children (45). Sex-specific physiological factors like hormones, fat distribution, and metabolism may contribute to differential responses between male and female offspring. Estrogen has shown to enhance gut barrier integrity and modulate immune responses, offering protective advantage for females against barrier disruption (46). Studies have also shown that females exhibit higher mucosal immune protection (47) and increased expression of genes involved in epithelial regeneration like Wnt/β-catenin signaling pathway (46, 48). This aligns with our observations where female offspring exhibited upregulation of tight junctions, integrins, and desmosome genes. The differences in fat deposition, metabolism, and tissue composition between male and female development may influence TC-ART drug exposure and its impact on the offspring. Together, all these factors including microbial diversity, lower levels of systemic inflammation, and relatively intact intestinal barrier function could have supported homeostasis in females and helped preserved the maintenance of body weight.

Overall, male rats exposed to *in utero* TC-ART through their mothers showed higher levels of transcriptional alteration of genes associated with intercellular communication and barrier integrity. Female rat offspring, despite the downregulation of gap junction and tight junction-related genes, showed activation of genes related to desmosomes, adherens junctions, and focal adhesion. These observations may suggest that transcriptional alterations in barrier, cell–cell adhesion, and cell–matrix interaction in male animals may reflect differences in adhesion- and signaling-associated pathways compared to females (**Figure 3A**). Moreover, transcriptional profiling provides a powerful and informative biomarker approach for chronic diseases, as differentially expressed mRNAs often correlate with corresponding protein changes and reflect underlying biological pathways. Importantly, transcriptional alterations may emerge early, enabling the identification of susceptible individuals or populations before overt clinical manifestation (49, 50). The increased intestinal permeability has been associated with translocation of luminal content, which may contribute to local and systemic inflammatory responses. These changes in the intestinal genes may also suggest that the sex-dependent outcomes in offspring could have been affected by sex-dependent hormones, physiology, or impact on the microbiome seeded during the early development of these animals.

In conclusion, our findings suggest that maternal TC-ART exposure has the potential to induce sexually dimorphic effects on intestinal permeability, gut mucosa-associated immune responses, villous morphology, and overall weight gain in offspring. Male offspring appear more susceptible to intestinal inflammation and barrier dysfunction, potentially contributing to their observed weight reduction. Females, while displaying structural ileal changes, may maintain homeostasis through alternative immune, microbiome, and adhesion mechanisms.

## Data Availability

The raw data supporting the conclusions of this article will be made available by the authors, as per US-Food and Drug Administration policies.
